# Family Centered Approach in Primary Health Care: Experience from an Urban Area of Mangalore, India

**DOI:** 10.1155/2015/419192

**Published:** 2015-01-27

**Authors:** Siddharudha Shivalli, J. P. Majra, K. M. Akshaya, Ghulam Jeelani Qadiri

**Affiliations:** ^1^Department of Community Medicine, Yenepoya Medical College, Yenepoya University, Mangalore, Karnataka 575018, India; ^2^Department of Community Medicine, BPS Government Medical College for Women, Khanpur Kalan, Sonepat, Haryana 131305, India; ^3^Department of Hospital Administration, Yenepoya Medical College, Yenepoya University, Mangalore, Karnataka 575018, India

## Abstract

*Introduction*. “Health for All” still eludes public health experts despite many approaches to prevent disease and promote health among urban poor. Several key illness factors lie beyond the conventional healthcare boundaries.* Objective*. To examine the effectiveness of family centered approach (FCA) in addressing health and related issues in an urban area of Mangalore, India.* Method*. A longitudinal study was conducted in* Bengre*, an outreach centre of Mangalore from June 2011 to November 2013. Family folders were created with pertinent details. Demand generation and health education activities were conducted through two female community health link workers. An FCA package was implemented by medical and nursing interns, under supervision, to address the priority issues. Effect was assessed by comparing their practices and service utilization before and after the study.* Results*. About 809 families participated in this study. Social, cultural, and religious factors were responsible for viciousness of malaria and maternal and child health issues. FCA improved their perceptions and practices towards health and related issues. Significant (*P* < 0.05) and sustained hike in service utilization was evident.* Conclusion*. FCA exposes key illness factors beyond the conventional care, eases need based healthcare implementation, and provides feasible and enduring solutions. Community involvement makes it more practicable.

## 1. Introduction

Urbanization is one of the leading global trends of the 21st century that has a significant impact on health. By 2050, over 70% of the world's population will live in cities. Today's cities and those of tomorrow are facing a triple threat: infectious diseases like HIV/AIDS, TB, and pneumonia; diarrhoeal diseases; noncommunicable diseases like asthma, heart disease, cancer, and diabetes; and violence and injuries, including road traffic injuries [1]. As per Census 2011, population of India has crossed 1.21 billion with the urban population at 377 million which is 31.16% of the total population. Urban areas provide great opportunities for individuals and families to prosper and can provide a healthy living environment through enhanced access to services, culture, and recreation. These positive aspects of city life attract people to come to and stay in urban areas [2]. In India such employment-driven migration is mainly from the “relatively less developed” states to large metropolises and other large cities, wherein the migrants get absorbed in low-paid jobs in the unorganized sectors [3]. These often settle in places which lack basic public services and hence are at risk of wide spectrum of health and related problems.

Contrary to the proximity of urban poor to healthcare facilities, their access and use is severely limited. Barriers are identified at both provider and beneficiary levels. Many approaches such as free or subsidized medical care, patient health cards, and incentive schemes have been tried to prevent diseases and promote health among urban poor. But the goal of “Health for All” still eludes the public health experts more so in developing countries like India. The concept of social determinants of health evidently underlines the existence of key illness factors beyond the conventional boundaries of primary care [4]. Hence, public health interventions must go beyond stethoscope and medicines and adopt a holistic approach to address health and related issues.

Family is reckoned as the unit of society and living and therefore should be the unit of illness. Family has been variously treated as an independent, dependent, and intervening variable, as a participating, predisposing, and contributory factor in the aetiology, care, and treatment of both physical and mental illness, and also as a basic unit of interaction and transaction of healthcare [5]. Family centered healthcare delivery could have a greater impact on addressing health and related issues and healthcare service utilization. It goes beyond providing care to individual patient to seeing them as being embedded in a family and providing services on that basis [4]. Family centered approach (FCA) is an attempt to draw two areas normally considered only as part of the “social determinants of health” background, education, and family welfare into the foreground of primary healthcare practice [6]. The core principles of family centered care are [7–13] as follows:

- treating clients and their families with dignity and respect,
- opening communication channels with clients and families,
- building up the strengths of client and family and promoting partnership between them,
- viewing client and family members as individuals and as members of a family and a community,
- regarding family as a key source of information about their relatives' and their own needs,
- tailoring the services to fit family needs and preferences and also ensuring that services are apt for a family's culture and traditions.

### 1.1. Objective

The aim of this paper is to examine the effectiveness of family centered approach (FCA) in addressing health and related issues in an urban area of Mangalore.

## 2. Method

### 2.1. Study Setting

**Figure 1:**
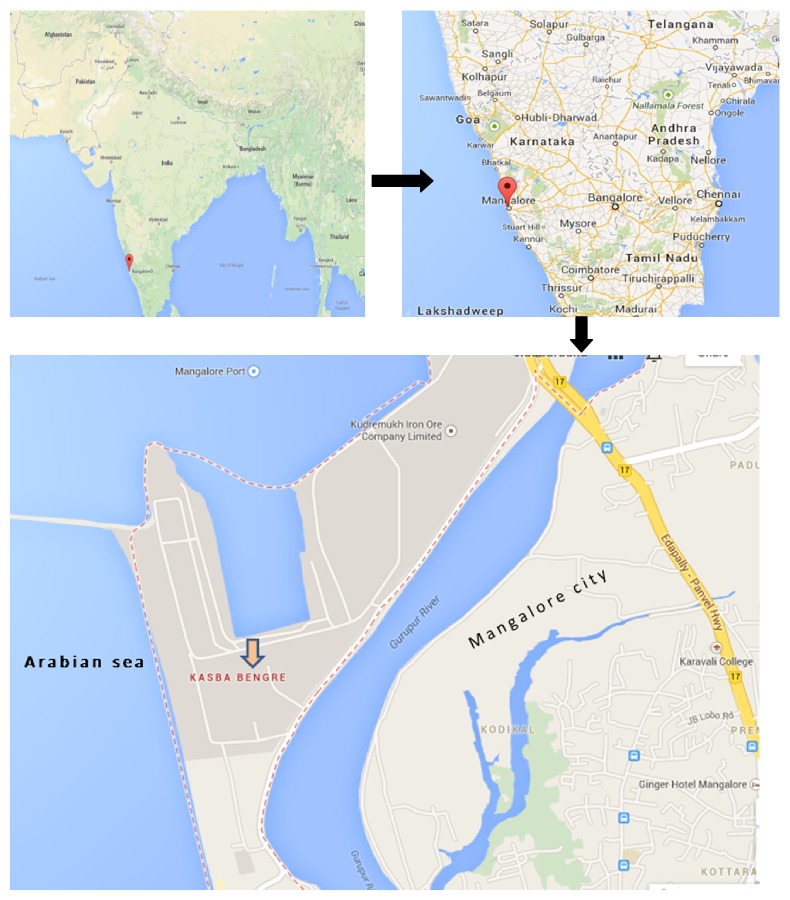
Bird's view of the study area* Bengre* in Mangalore City, India [14].

Mangalore is a coastal city located in Karnataka State, India. It is bordered by the Arabian Sea on west and Western Ghats on east.* Bengre* is an outreach centre located in municipal ward number 60 of Mangalore City (Figure 1). Situated between* Gurupura* River and the Arabian Sea,* Bengre* is considered as an underserved area, settled mostly by poor people belonging to labour class. It is inhabited by more than 800 families with a population of 5,368. Department of Community Medicine, Yenepoya Medical College, Mangalore, is providing primary care services in the study area on alternate days.

### 2.2. Approach

**Figure 2:**
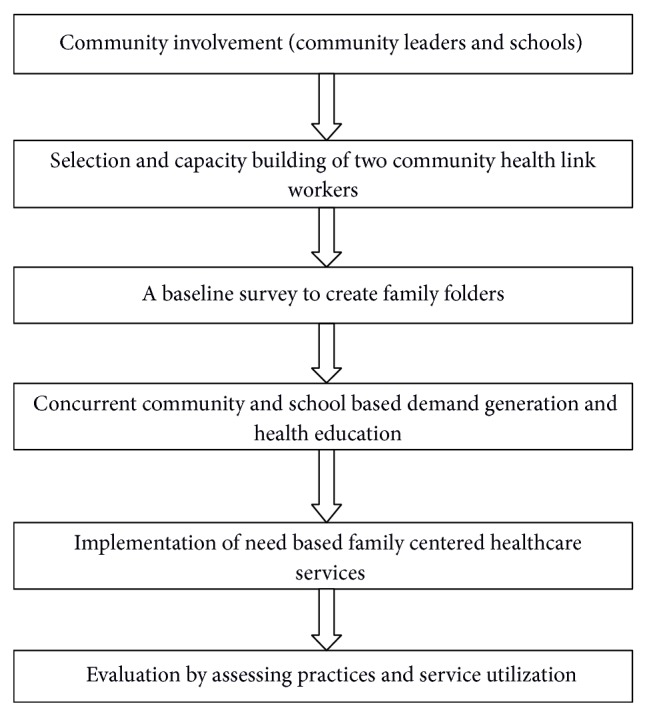
An outline of family centered approach adopted in this study.

This longitudinal study was conducted from June 2011 to November 2013 (Figure 2). Firstly, local community leaders and school teachers were approached and enlightened about the long term benefits of family centered primary healthcare and importance of community involvement. Two women, residents of the same locality, in the age group of 25 to 45 years and literate with formal education up to class eight were selected as community health link workers. Selection criteria were the same as for the Urban Social Health Activist (USHA) under the National Urban Health Mission which is in early phase of implementation across the country [15]. They were trained for community mobilization and involvement so that the implementation of FCA is facilitated.

A baseline survey was conducted in first 6 months to create family folders and prioritize the issues. Each family folder consisted of sociodemographic profile, maternal and child health details including family planning, environment and microdistrict details, morbidity and mortality in last one year, knowledge of common diseases, and health seeking behaviour of the family. Family folder was validated by 3 research experts and pretesting was done on 10 families. All school children were screened for common nutritional and health problems. Data so gathered was analyzed to prioritize health and related issues.

Concurrent community and school based demand generation and health education activities were carried out. A service package was designed to be implemented by medical and nursing interns, under the supervision of authors, to address the priority issues. It consisted of the following:

- reviewing the family folder of patient attending outpatient clinic and efforts to analyze the key illness determinants and offer feasible and long lasting solutions, apart from medicines, on case to case basis,
- opportunistic screening of person/s accompanying the patient attending outpatient clinic and imparting pertinent health education,
- weekly health education and behaviour change communication (BCC) sessions for women to enhance awareness of common diseases, maternal and child health, and importance of environmental and personal hygiene; a microplan preparation to cover the study area by two teams; and female community link workers to facilitate women mobilization and involvement,
- hands on training for women about hand washing, water filtration and safe storage, exclusive breast feeding and complementary feeding, use of oral rehydration salt (ORS), environmental hygiene, and related issues.

Need based family centered healthcare services were imparted from January 2012 to November 2013 keeping family and economic status in the background.

Effect of FCA was assessed by the authors by comparing practices and service utilization before and after the study. Feedback was collected from a subsample (10% of the total population) in the study area selected by systematic random sampling with the help of a validated and pretested interview schedule. Service utilization was assessed by comparing the monthly patient turnout before and after the FCA.

### 2.3. Statistical Analysis

Data so gathered was analyzed using Statistical Package for the Social Sciences (SPSS) Inc., Chicago, USA, Version 16.0. Continuous variables were expressed in mean and standard deviations and categorical variables were expressed as number and percentages. Wilcoxon signed-rank and chi-square tests were used to assess the difference in service utilization and key outcome variables before and after FCA in the study area. All tests were two-tailed and *P* < 0.05 was considered as significant.

### 2.4. Ethical Issues

University Ethics Committee approved the study protocol and necessary permissions were sought from the district health authorities. Informed written consent was taken from the head or an adult member of the family for voluntary participation.

## 3. Results

A total of 809 families participated in this study. Most (96.3%) of the families belonged to Islam religion and most of them were either fishermen or sailors (Table 1).* Bidi* (Indian cigarette filled with tobacco flake and wrapped in a leaf) rolling was the predominant occupation among women (46.7%). Nuclear family system was in vogue (64%) with an average family size of 6.7 (±3.2). Almost half (49.9%) of the families were below poverty line as per the state government ration card/public distribution system. Almost all (97.2%) the families had a household latrine; however, one fourth of them were insanitary. Children under five years constituted 10.2% of the total population with a child sex ratio of 858 girls/1000 boys. Private sector was the main source of health services in the study area. Analysis of family folders and demand generation activities revealed malaria and maternal and child health problems as priority issues.

### 3.1. Malaria

Despite satisfactory awareness (71.4%), malaria prevalence was high in the study area. In fact, entire district (*Dakshina Kannada*) is classified as highly endemic with annual parasite incidence (API) of 2 to 5 [16]. It is largely attributed to geographical tenure and construction activities, complemented by poor environmental conditions.

Mosquito breeding was observed in 83.1% of the houses and housefly (55.6%) and rodents (52.4%) were the problems in more than half of the families. Use of mosquito repellents (liquidator and coils) and seeking medical care were the main antimalarial measures implemented in the study area. Cost effective and long lasting measures like simple environmental engineering, bed nets (21.2%), and wire meshing of windows (19.8%) were completely overlooked. The same was explained when patient sought treatment at our clinic and in health education sessions. At the end of the study 62% and 58% of the families were using bed nets and had screened their houses with wire mesh, respectively (*P* < 0.001). The number of reported malaria cases reduced to 90 from 150/1000 population per year.

### 3.2. Family Planning

Nearly half of the eligible couples had their first child within one year of marriage. Awareness of various family planning methods was poor (21.4%) (Table 2). Only 12% of 1409 eligible couples had ever adopted family planning. This was very low when compared to district average (51%) [17]. Copper-T and tubectomy were predominant ways of family planning among those who ever practiced. Average family size in the study area was far higher than state average. Prejudiced religious views and misconceptions were the reasons for poor acceptance and practice of family planning. At the end of the study nearly 32% of the eligible couples were practicing any method of family planning.

### 3.3. Maternal Health

Though ante-, intra-, and postnatal service utilization was high (>90%), dietary intake was below par among half of the pregnant and lactating women (Table 3). Many misconceptions like “fear of big baby,” “hot and cold concepts of food,” and so forth were prevalent in the study area and were successfully addressed by health education. Mothers-in-law and husbands of the pregnant women and other decision makers in the family were involved in the same to address the dietary misconceptions and help the pregnant women to enhance their dietary intake. At the end of FCA, almost three fourths of them (71%) could take one extra meal during pregnancy and lactation.

### 3.4. Child Health

Status of child health interventions such as essential obstetric and postnatal care to mother, hospital delivery, exclusive breastfeeding for 6 months, and immunization was admirable (Table 3). However, status of vitamin A prophylaxis (40%) was unsatisfactory. Repeated episode of acute diarrhoea was the main child health issue. Poor hygienic and improper complimentary feeding practices were the reasons for acute diarrheal diseases among children. Nearly half of the under five children (43%) had suffered from diarrhoea in the last fifteen days from the date of survey. Apart from ORS use, we stressed on personal and hand hygiene and adequacy of complement feeds through hands on training for mothers. At the end of FCA, 29.2% of the under five children, in randomly selected families for the feedback, had diarrhoea in the last fifteen days from the date of survey.

More than one third of the adults were current tobacco users. It was due to easy availability as half of the families were involved in* bidi* rolling. Theme based health education sessions were conducted to address the same.

**Figure 3:**
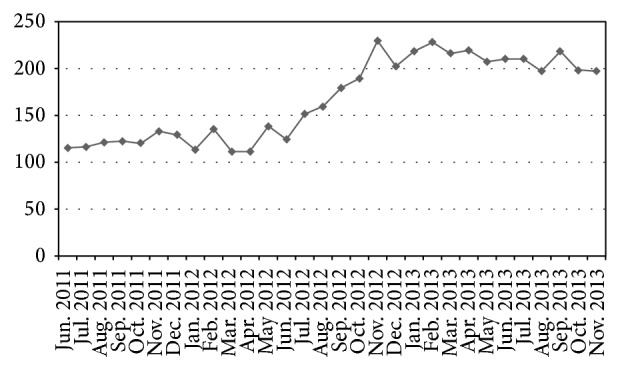
Average monthly outpatient turnout in the study area (Jun. 2011–Nov. 2013) (family centered approach was initiated in Jan. 2012).

Statistically significant (*P* < 0.05) and sustained increase in healthcare service utilization was observed after 6 months of implementation of family centered services (Figure 3). However, behaviour change is a long term process which requires sustained efforts and regular reinforcements to see its significant impact in terms of decline in disease morbidity and mortality.

### 3.5. Capacity Building

Capacity building of medical and nursing interns as future family physicians and community health nurses was an important collateral benefit owing to their extensive involvement at all the stages.

### 3.6. Feedback from Beneficiaries

A total of 106 families, selected by systematic random sampling, gave their feedback. Over three fourths of the families had attended at least one health education session and more than half of these had attended 3 or more. More than 80% of them found health education sessions very useful in terms of increase in the awareness, simple preventive measures, and availability of free treatment. Even illiterate people could enhance their knowledge level through these sessions. When beneficiaries were asked to recall the topics covered in health education sessions, most of them recollected sessions on vector borne diseases (especially malaria), personal hygiene, diarrhoea, diabetes, and cardiovascular diseases.

Communication through* Beary* dialect and use of simple terms during health education were the predominant suggestions to improve.

## 4. Discussion 

All the members of a family share common physical, social, and biological environment which has direct impact on their health. Therefore, family as a unit is very important for providing comprehensive health services. This study was done in a typical urban marginalized community with nearly half of the families below poverty line.

FCA was an effective strategy to explore and address the key illness and health related factors beyond the boundaries of conventional primary care. Involving local community leaders and schools was crucial to the enhancement of community involvement and accountability. Endorsing community involvement and demand generation through health education would make FCA more feasible. Two community link health volunteers were the torchbearers in this approach and to larger extent bridged the gap between community and the healthcare system.

This study and others [18–22] suggest that considerable proportion of people living in endemic area are familiar with the term “malaria,” its symptoms, and mode of spread. Diverse factors such as age, gender, education, and economic status, personal experience of malaria, transmission level, and treatment availability and accessibility have been pointed out for knowledge variations. Use of insecticide treated bed nets is one of the cost effective interventions to curb malaria and other vector borne diseases [23–25]. Corroborating to our findings, lower bed net knowledge and use are reported in studies from New Delhi, India [22], Haiti [26], Turkey [27], Ethiopia [28], and Iran [29]. However, higher bed net knowledge was reported in Bangladesh [30], Nepal [31], and Ghana [32]. In FCA, targeted health education sessions stressed on bed net use, meshing the windows, and clean immediate surroundings. In the end, significant rise in implementation of the same and fall in malaria incidence were evident.

Due to profound hormonal changes and minor ailments in first trimester, there would be an expected loss of appetite among pregnant women. Although this effect gets blunted as the pregnancy advances, most women continue to take deficient diet because of myths and taboos. This is corroborated by our and many studies across the globe [33–36]. Apart from health education of pregnant women, involvement of their mothers-in-law and husbands could address this issue to a larger extent. Studies by Story and Burgard [37] and Martin et al. [38] also recommended partner and decision maker's involvement in antenatal care for greater net impact on maternal health behaviors. FCA offers ample opportunities for the same and also to address the prevailing misconceptions. Similar strategies were used to enhance family planning use and to address child health issues.

Family folder system is an effort towards development of FCA in addressing health and related issues and to organize healthcare services. It provided a framework to plan FCA and theme based health education activities. Data from family folders was of great use for assessing community health needs, clients' segmentation and prioritization, preparation of efficient work schedule, and tracking the clients for continuity of services. Annual Report of Christian Medical College, Ludhiana, India, 1997 [39], and Majra and Acharya [40] endorsed the family folder system and described it as wonderful health management information system tool, if implemented sincerely. Enhanced service utilization (Figure 3) and favourable practices were clearly evident in this study (Table 4). However, acceptance and practice of family planning and quitting tobacco were relatively difficult to address and require continuous efforts to foresee the impact.

According to Abraham and Moretz [41], patient- and family-centered care applies to patients of all ages, and it may be practiced in any healthcare setting at all levels of healthcare organization. In fact, patient- and family-centered paediatric care has become the “gold standard” in paediatric care. It is further supported by better outcomes in terms of quality, safety, and patient/family satisfaction [42–44]. In patient- and family-centered care, patient and family knowledge, values, beliefs, and cultural backgrounds are incorporated into the planning and delivery of care. Patients and families receive timely, complete, accurate, and unbiased information in order to effectively participate in care and decision-making [45]. Such an approach is essential to break the viciousness of health problems and offer feasible and long lasting solutions. FCA is a fundamental shift in the distribution of power to give patients and their families an active voice in their healthcare. It leads to better health outcomes, improved quality and safety, wiser allocation of resources, and greater patient, family, and staff satisfaction [46]. Hence, reorientation of all the healthcare providers and their capacity building and creating environment in the existing healthcare delivery system are the challenges ahead. Addressing the legal and ethical issues of sharing information of the patient with other family members is also needed.

Many aspects of FCA do not cost more money; they simply require a change in attitude and approach. It improves the quality and effectiveness of communication. It is proactive, rather than reactive. As a result, many problems are prevented, and others are handled before they grow out of control [47]. Family centered care is evidence based best practice [48]. Available evidence suggests that involving families hastens patient recovery, reduces reliance on healthcare services, reduces the rate of relapse, enhances medication compliance, and bolsters client interpersonal functioning and family relationships [49, 50].

Government of India in rural areas has integrated all the national health programs under the umbrella of National Rural Health Mission and the same is being emulated in urban areas under National Urban Health Mission [15]. However, grass root level workers are overburdened with updating of different registers to generate data and to report information to higher levels. Whatever data collected is seldom used for future planning and prioritization [30, 51]. Family folder system could be an excellent and easy way of integration of information and to strengthen the health information management system. Many medical colleges in India do have family folder system in their urban and rural field practice areas [52–54]. Such systems need to be implemented in full capacity and to be evaluated critically. Capacity building of medicos, nurses, and grass root level healthcare workers in this regard is required.

## 5. Conclusion

FCA in primary healthcare is an effective strategy to explore the key factors beyond the conventional care and offers feasible and long lasting solutions. Exploring sociocultural beliefs and perceptions is crucial to address and break the vicious cycle of health and related issues. Community link health volunteer is essential to facilitate community mobilization and involvement in this endeavour. Orientation and training of various healthcare personnel, that is, medical students, nurses and grass root level healthcare workers towards FCA is required.

## Supplementary Material

Family centered approach in primary health care: Experience from an urban area of Mangalore, India.

## Figures and Tables

**Table 1:** Key sociodemographic characteristics of the study population.

Characteristic	*N*	%
Total population	5,368	100
Total households	809	100
Religion		
Muslim	779	96.3
Hindu	26	3.2
Christian	4	0.5
Type of family		
Nuclear	518	64
Joint/extended	291	36
Number of reproductive age group women	1539	28.7
Number of eligible couples	1409	26.2
Under five children		
Male	295	5.4
Female	253	4.6
Source of health services for the family		
Public sector	186	23
Private/others	623	77

**Table 2:** Family planning (FP) status among eligible couples in the area before the implementation of family centered approach (*n* = 1409).

Study variable	Mean/*n*	SD/%
Mean age of marriage for women	18.6	1.2
Mean age of marriage for men	24.14	2.8
Mean age at 1st child for women	19.9	1.6
Mean age at 1st child for men	25.3	2.1
Average number of children	2.7	1.1
Spontaneous abortions	61	4.3%
MTPs^*^	78	5.5%
Knowledge of FP	302	21.4%
Ever practiced FP	178	12.6%

^*^Medical termination of pregnancy.

**Table 3:** Status of child health interventions in the study area before the implementation of family centered approach (*n* = 548).

Study variable	*n*	%
Birth registered	527	96.1
Mother received essential obstetric care	543	99.1
Mother received postnatal care	542	98.9
Delivery in a healthcare setup	528	96.3
Delivery by LSCS^*^	64	11.7
Low birth weight babies	73	13.3
Fully immunized children	499	91.1
Exclusively breast fed for 6 months	513	93.6
Vitamin A prophylaxis^#^	219	40.0

^*^Lower segment caesarean section. ^#^Children aged >9 months.

**Table 4:** Key study variables before and after the implementation of family centered approach (FCA) in the study area.

Study variable	Pre-FCA	Post-FCA^#^	*χ* ^2^	*P*
Proportion of houses using bed net (*n* = 809)	21.2	62	34.26	<0.001
Proportion of houses with wire meshed windows (*n* = 809)	19.8	58	30.67	<0.001
Incidence of malaria (per 1000 population)	150	90	17.045	<0.001
Proportion of eligible couples who ever practiced any method of family planning (*n* = 1409)	12.6	32	8.635	0.003
Proportion of pregnant and lactating women who ate one extra meal (*n* = 182)	52	71	7.623	0.006
Proportion of under five children with ADD in the last fifteen days (*n* = 548)	43	29.2	4.128	0.04

FCA: family centered approach.

^#^Post-FCA assessment was done on 106 families/households selected by systematic random sampling.

## Abbreviations

FCA: Family centered approach
BCC: Behaviour change communication
USHA: Urban social health activist.
