# Supplementary material for: Family Centered Approach in Primary Health Care: Experience from an Urban Area of Mangalore, India
Source: ScientificWorldJournal. 2015 Jan 27;2015:419192. doi: 10.1155/2015/419192 (PMC4322852; doi:10.1155/2015/419192)
Supplement: Supplementary file 1 — Family centered approach in primary health care: Experience from an urban area of Mangalore, India. [file 419192.f1.docx]

**Family centered approach in primary health care: Experience from an urban area of Mangalore, India**


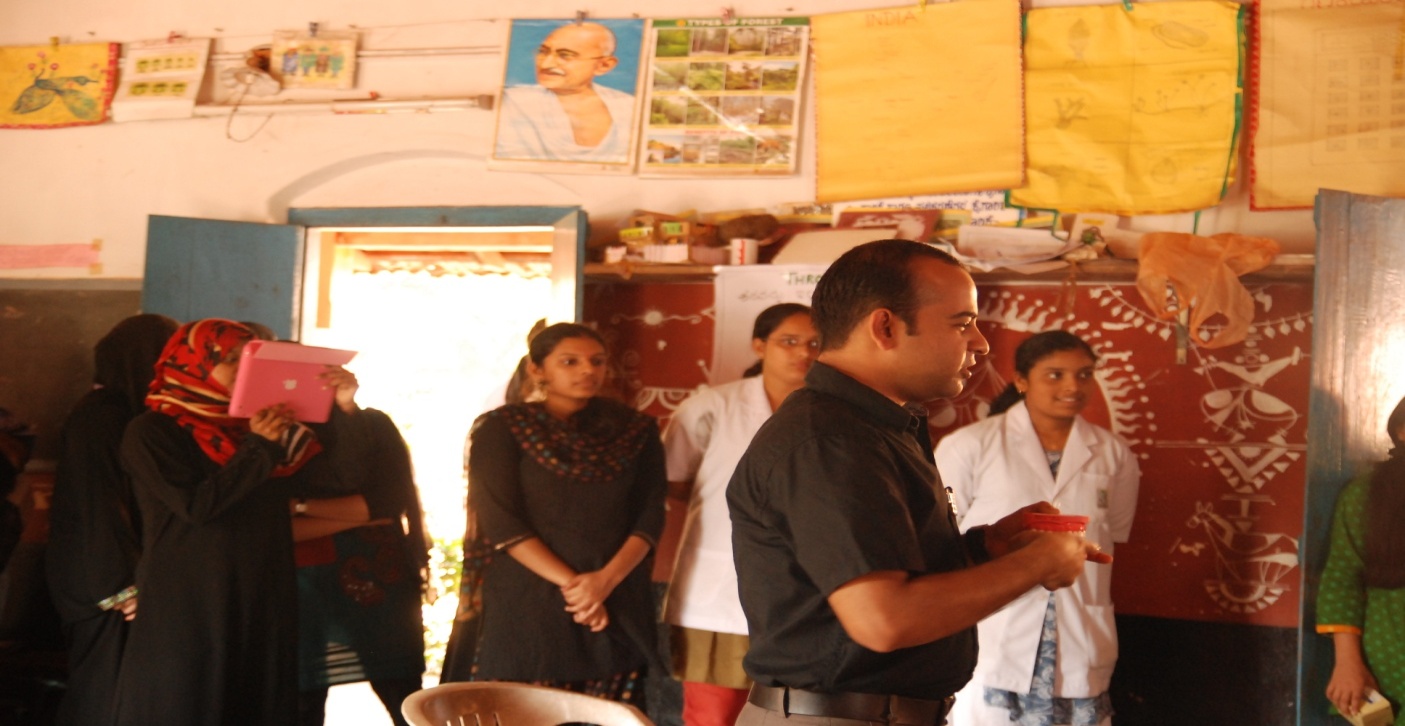


School based health education session about dental and personal hygiene by Dr.Siddharudha Shivalli


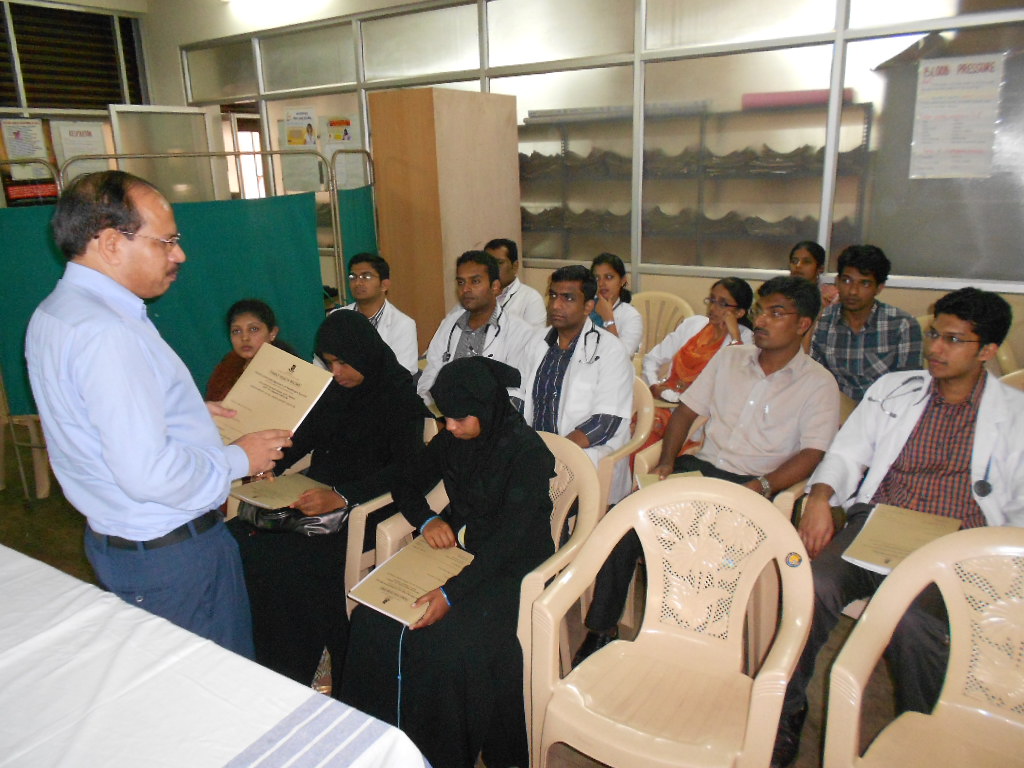


Orientation of Family centred approach to medical interns by Dr.JP Majra and the two women sitting in the front row were the community health link workers under this project


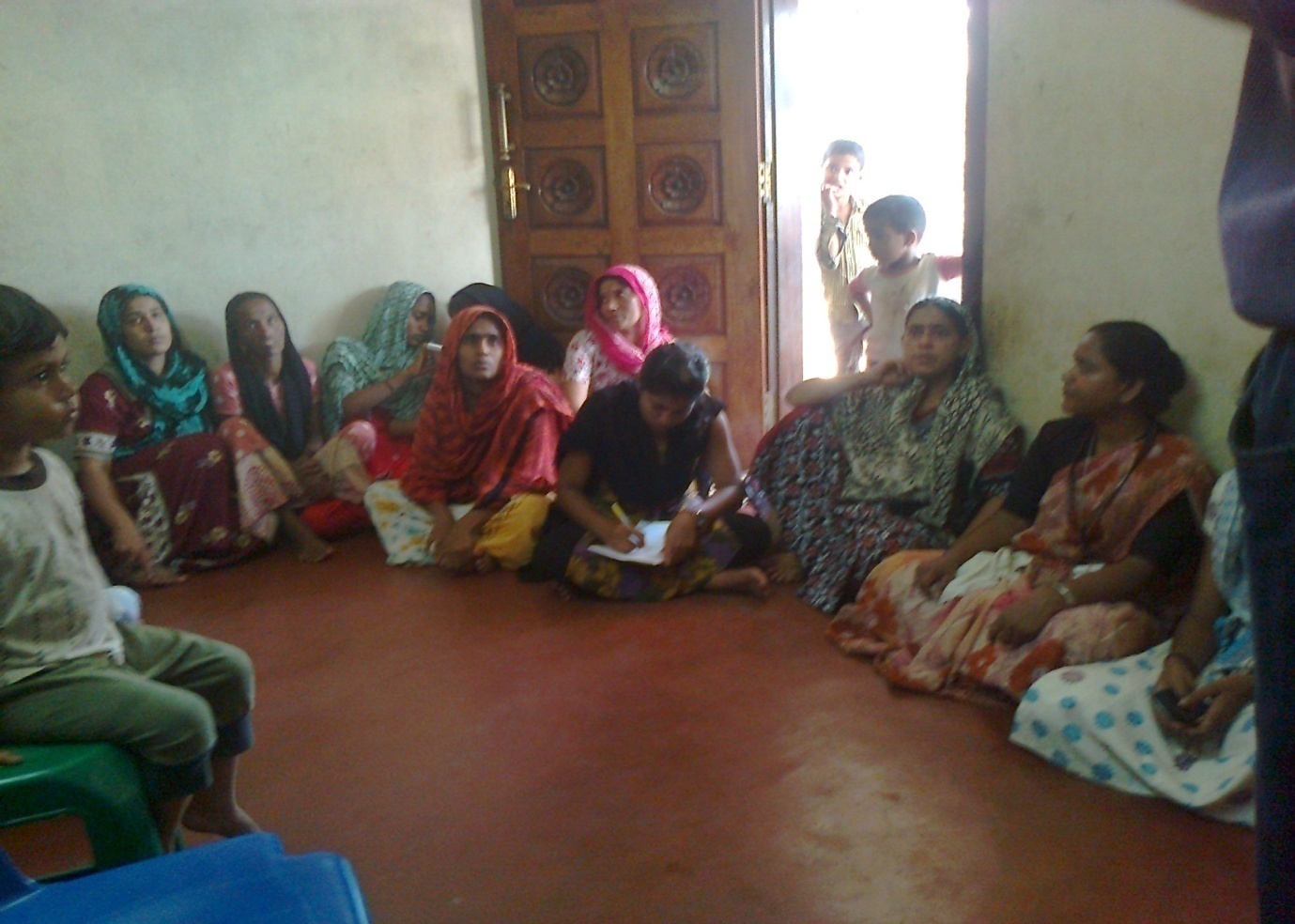


Beneficiaries of health education session held at *Bengre*


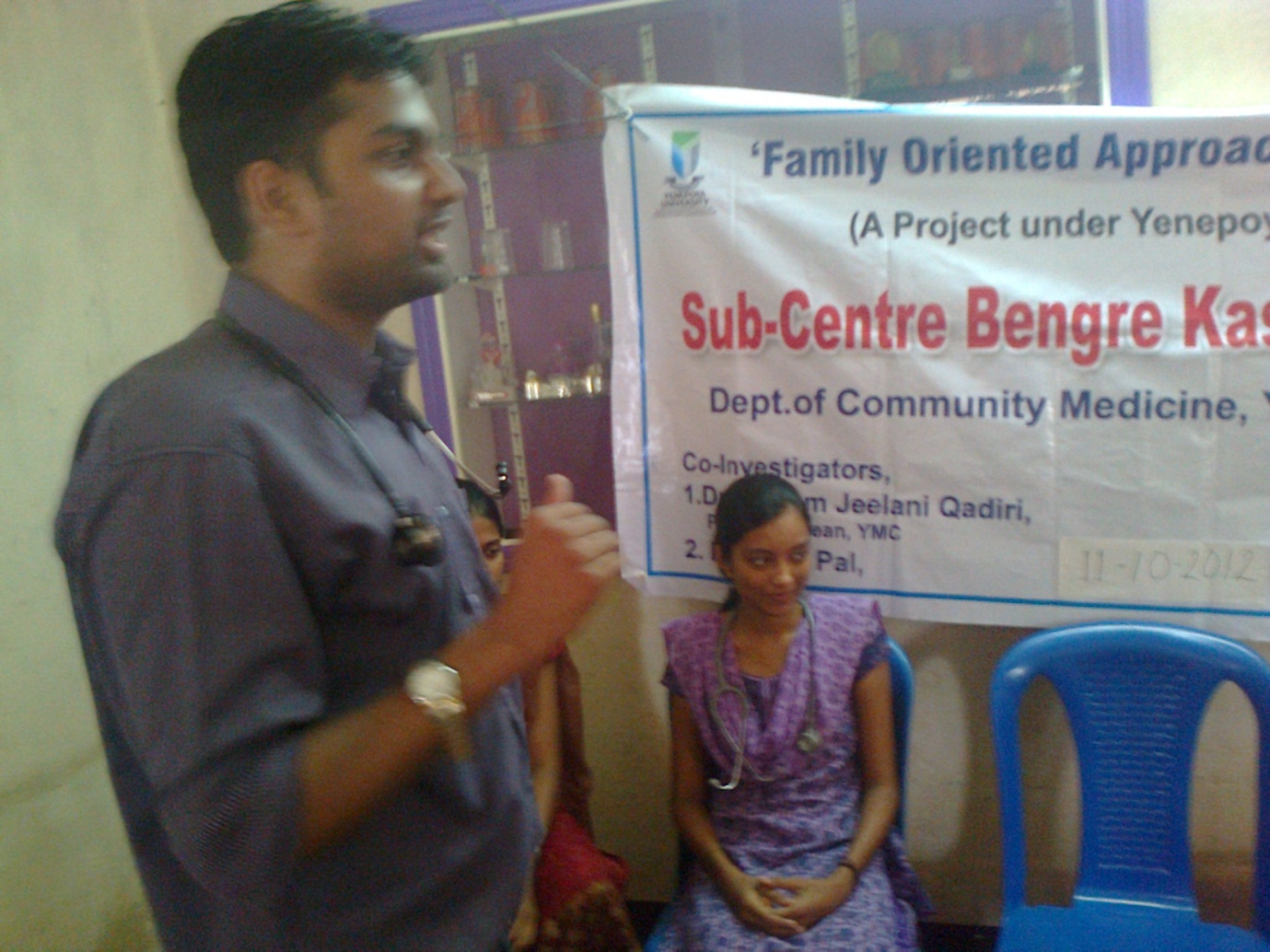


Medical intern addressing importance of antenatal care

We conducted a longitudinal study in *Bengre*, an outreach centre of Mangalore from June 2011 to November 2013 to examine the effectiveness of family centered approach (FCA) in addressing health and related issues. Family folders were created with pertinent details. Demand generation and health education activities were conducted through two female community health link workers. An FCA package was implemented by medical and nursing interns, under supervision, to address the priority issues.

About 809 families participated in this study. Social, cultural and religious factors were responsible for viciousness of malaria and maternal and child health issues. FCA improved their perceptions and practices towards health and related issues. Significant and sustained hike in service utilization was evident. FCA exposes key illness factors beyond the conventional care. Eases need based healthcare implementation and provides feasible and enduring solutions. Community involvement makes it more practicable. Capacity building of medicos, nurses and grass root level healthcare workers in this regard is required.
